# Editorial: Pathobiological defects in sensorineural hearing loss: from identification to rescue

**DOI:** 10.3389/fnmol.2025.1654457

**Published:** 2025-07-29

**Authors:** Emanuele Bernardinelli, Laura Astolfi, Konstantina M. Stankovic

**Affiliations:** ^1^Institute of Pharmacology and Toxicology, Paracelsus Medical University, Salzburg, Austria; ^2^Bioacoustics Research Laboratory, Department of Neuroscience-DNS, University of Padova, Padova, Italy; ^3^Stanford University Department of Otolaryngology and Head and Neck Surgery, Stanford, CA, United States; ^4^Stanford University Department of Neurosurgery, Stanford, CA, United States; ^5^Stanford University Wu Tsai Neurosciences Institute, Stanford, CA, United States

**Keywords:** hearing loss, prevention, diagnosis, sensorineural hearing loss (SNHL), rescue

Hearing loss is the most common sensory impairment, affecting around 1 in 1,000 newborns (Koffler et al., 2015; Morton and Nance, 2006). This condition profoundly affects the development of language skills and impacts cognitive development (Niparko et al., 2010; Tomblin et al., 2015). Timely diagnosis is essential, yet hearing loss can lead to permanent effects even if identified. The causes of hearing impairment are complex and multifactorial, involving genetic mutations, ototoxicity and environmental influences (Korver et al., 2017; Le et al., 2017; Fink, 2024). Genetic factors play an essential role in hearing loss, with mutations in genes such as *SLC26A4, GJB2*, and *POU3F4* being among the primary causes of hereditary deafness (Busi et al., 2012; Bernardinelli et al., 2022, 2025b; Sklenar et al., 2025). Furthermore, mitochondrial DNA mutations, such as A1555G and T961G in the *MTRNR1* gene, have been associated with aminoglycoside-induced deafness (Guaran et al., 2013; Castiglione et al., 2013). The Kir4.1 potassium channel, encoded by the *KCNJ10* gene, is essential for maintaining endocochlear potential, and its dysfunction is associated with various syndromes (Fracaro et al., 2024). Understanding these genetic factors is crucial for developing targeted therapies and enhancing genetic counseling.

The auditory system comprises highly specialized cell types that convert sound waves into electrical impulses (Jeong et al., 2022). The development and homeostasis of the hearing organ involve numerous molecular players, including transcription factors, ion channels, transporters, structural proteins and enzymes. Any alteration of these tightly regulated molecular mechanisms can lead to varying degrees of hearing impairment (Schwander et al., 2010). Many hearing defects originate during embryogenesis when structural malformations occur (Sennaroglu and Bajin, 2017), which result in permanent alterations to the structure of the inner ear and cannot be rectified once occurred.

Current therapeutic options for hearing loss include hearing aids and cochlear implants, but not all patients benefit from these (Le et al., 2017). As a result, research is focusing on strategies to address developmental abnormalities leading to hearing loss (Li et al., 2013; Wang et al., 2024). In certain instances, a pharmacological intervention can be considered to restore the function and expression of a specific target protein (Bernardinelli et al., 2025a). Additionally, tissue engineering and 3D printing are transforming auricular reconstruction for patients with microtia, enhancing aesthetics and auditory function (Hellies et al., 2025).

The latest advancements in hearing loss research have revealed various aspects of auditory function and dysfunction. For instance, Bieniussa et al. analyzed how Stat3 contributes to the development and differentiation of mouse hair cells. Their findings revealed that Stat3 knockout in outer hair cells (OHCs) led to increased distortion product otoacoustic emissions (DPOAE) levels and higher hearing thresholds. These defects were associated with increased pro-inflammatory activity, including up-regulation of the NF-kB pathway and increased cytokine production. When Stat3 was knocked out in supporting cells, similar hearing defects were observed and they were linked to cytoskeletal instability. The upregulation of pro-inflammatory pathways and cytoskeletal instability in Stat3-deficient mice provides new targets for therapeutic interventions. This research enhances our understanding of cochlear homeostasis, highlighting the potential of modulating Stat3 activity as a novel approach to preserving hearing function.

Advancements in imaging techniques have enhanced our ability to visualize the structures and functions of the cochlea. Schulz-Hildebrandt et al. describe an advancement of optical coherence tomography (OCT). They introduced a dynamic imaging component, enabling real-time tracking of metabolic activity by monitoring ATP-dependent intracellular motion. This innovation reduces acquisition time and minimizes potential damage to the cochlear structure while allowing visualization of individual cochlear cells (Iyer et al., 2016). This technical breakthrough has far-reaching implications for both research and clinical applications. The ability to monitor ATP-dependent intracellular motion non-invasively opens new possibilities for studying cochlear pathophysiology *in vivo*. This technology could revolutionize our approach to diagnosing and treatment by providing unprecedented insights into cellular dynamics within the living cochlea.

The role of cellular stress in hearing loss has also been a recent research focus. Li et al. explored the role of endoplasmic reticulum stress (ERS) in SNHL. The accumulation of misfolded proteins within the ER triggers the unfolded protein response (UPR), which can activate inflammatory pathways, oxidative stress responses, autophagy and cell death, eventually contributing to the development of SNHL. The review discusses potential pharmacological interventions to enhance cell survival by modulating ER stress. This work provides a cohesive framework by clarifying the intricate role of ERS and UPR in several types of hearing loss. They discuss potential pharmacological interventions targeting ERS pathways, such as Salubrinal, Pitavastatin and Tauroursodeoxycholic acid (TUDCA), opening promising new avenues for developing therapeutic strategies that could revolutionize the treatment of SNHL.

Focusing on synaptic transmission in the auditory pathway, Chen et al. investigated otoferlin, a protein crucial for synaptic function in auditory hair cells. By introducing mutations in key amino acids within the C2F domain, they demonstrated that the mutated otoferlin protein accumulated in the Golgi apparatus, failing to reach the basolateral membrane of inner hair cells. Although calcium influx was not affected, calcium-triggered exocytosis of synaptic vesicles was significantly impaired. The researchers identified a key mechanism in auditory signal transduction by showing that specific mutations in this domain of the protein otoferlin can cause severe deafness while leaving hair cell morphology unchanged. The accumulation of mutated otoferlin in the Golgi apparatus, coupled with impaired calcium-triggered exocytosis, provides a link between protein trafficking, synaptic function and hearing ability. This study therefore provides novel insights into the mechanisms of synaptic transmission within the auditory system, supporting the ongoing otoferlin gene therapy trials to restore auditory function.

The development of the inner ear is a tightly orchestrated process and, as a prevalent pathomechanism leading to hearing loss, it is an area of intense study. Ito et al. explored the role of the anion exchanger pendrin (Slc26a4) in the development of the bony labyrinth and in the otoconial mineralization. Slc26a4-deficient mice exhibited incomplete partition of the cochlea, enlarged vestibular aqueduct and impaired endolymphatic fluid reabsorption, associated with larger otoconia in the utricle and a near absence in the saccule, leading to the commonly observed vestibular dysfunction. These findings underscore the essential role of specific genes in the proper formation and function of the inner ear, bridging the gap between genetic mutations and phenotypic outcomes, and paving the way for potential therapeutic interventions targeting the SLC26A4 pathway.

In conclusion, recent research has markedly advanced our understanding of the molecular and cellular mechanisms that underlie hearing loss. Studies on Stat3, otoferlin and ER stress have provided valuable information on the genetic and biochemical factors influencing auditory function. In addition, innovations in cochlear imaging and investigations into inner ear development are promising to improve diagnostic and therapeutic strategies. Although significant progress has been made, further research is necessary to refine treatment approaches and develop effective interventions for preventing and managing hearing loss. Hearing disorders' complexity necessitates a multidisciplinary strategy that integrates genetics, molecular biology, physiology, optical imaging and clinical medicine to tackle this common sensory impairment. The studies presented in this Research Topic collectively represent significant milestones in hearing research, each contributing unique and valuable insights into the intricate mechanisms that underlie auditory function and dysfunction, while shaping future diagnosis and therapy.
